# Effects of Selenium Supplementation in Patients with Mild Cognitive Impairment or Alzheimer’s Disease: A Systematic Review and Meta-Analysis

**DOI:** 10.3390/nu14153205

**Published:** 2022-08-05

**Authors:** Meire Ellen Pereira, Júlia Vicentin Souza, Maria Eduarda Andrade Galiciolli, Fernanda Sare, Giovanna Scorsin Vieira, Isabeli Lopes Kruk, Cláudia Sirlene Oliveira

**Affiliations:** 1 Instituto de Pesquisa Pelé Pequeno Príncipe, Rua Silva Jardim 1632, Curitiba 80250-060, Brazil; 2 Faculdades Pequeno Príncipe, Avenida Iguaçu 333, Curitiba 80230-020, Brazil

**Keywords:** selenium, neurodegenerative disease, Brazil nut, oxidative stress

## Abstract

Elevated levels of oxidative stress could cause and aggravate Alzheimer’s disease (AD). Selenium (Se) is a trace element with antioxidant and anti-inflammatory activity with neuroprotective effects. To evaluate the effects of Se supplementation in patients with AD or mild cognitive impairment (MCI) through a systematic review and meta-analysis, data were searched and collected from four electronic databases, including clinical trial studies published until December 2020, following the PRISMA guidelines. Statistical analysis was performed by RevMan, and the risk of bias was assessed using the Rob 2 tool. A total of 1350 scientific papers were collected, and following evaluation 11 papers were included in the systematic review and 6 of these were used in the meta-analysis. Studies that evaluated only Se supplementation observed an improvement in Se levels, glutathione peroxidase (GPX) activity, and in some cognitive tests in MCI patients; similarly, improvement in Se levels and mini-mental score was also observed in AD patients. Regarding supplementation of Se plus other nutrients, improvement in cognitive tests was observed in both AD and MCI patients. Therefore, Se supplementation is a good alternative for patients with AD and MCI for improving Se levels and GPX activity. More detailed studies are required to further evaluate the effects of Se on the cognitive deficit and oxidative stress associated with AD and MCI.

## 1. Introduction

Selenium (Se) is a trace element that can be found naturally in inorganic (e.g., selenite and selenate) or organic (e.g., selenocysteine and selenomethionine) chemical forms [1,2,3,4,5]. Se is essential for human health because it is part of selenoproteins, which participate in antioxidant and anti-inflammatory pathways [1,6,7]. Se is pivotal for human development and plays a significant role in the central nervous system [1,6]. Several studies in rodents have demonstrated that the depletion of some selenoproteins, such as selenoprotein P (SELP), causes irreversible brain damage, predisposition to seizures, impaired motor coordination, and cognitive decline [7,8,9]. Interestingly, Rueli et al. [10] demonstrated that SELP is significantly increased in the choroid plexus and cerebrospinal fluid of patients with Alzheimer’s disease (AD); on the other hand, Garlet et al. [11] observed a decrease in blood glutathione peroxidase (GPX) activity in AD patients. However, the precise roles of Se and selenoproteins in AD are still not well established.

Described in 1907 by the German neuropathologist Alois Alzheimer, AD is a progressive neurodegenerative disease that manifests itself with reduced cognitive ability and memory loss, being one of the most common forms of dementia. It is characterized by the existence of neurofibrillary tangles containing β-amyloid peptide and tau protein in the pathological brain tissue, leading to neuronal degeneration [7,12,13]. Moreover, its pathophysiology is related to the large production of reactive oxygen species, inflammatory markers, decreased antioxidant agents, and brain metabolic changes [7,12,13,14].

Mild cognitive impairment (MCI) can be defined as a decline in cognition beyond what is expected in normal aging. This impairment cannot yet be characterized as dementia, but it is associated with lower quality of life, decreased independence, relationship problems, and greater medical needs [15,16]. Therefore, depending on the underlying cause, the clinical condition of patients with MCI may improve, stabilize, or progress to dementia [17,18].

Dietary supplementation composed of adequate nutrients may be associated with a decreased risk, not only for developing AD but also other dementias [19,20]. Several nutrients, such as vitamins C, D, and E, vitamins of the B-complex, and omega-3, are associated with improvements in the symptoms of AD and MCI [21,22,23]. Similarly, Se has been studied as a trace element with possible benefits in neurodegenerative diseases and has been analyzed as a potential agent for preventing the development and progression of AD and MCI [24,25].

Studying the relationship between Se intake and the maintenance of brain function can help to understand the benefits of Se in AD and MCI. Therefore, the objective of this systematic review and meta-analysis was to evaluate the effects of Se supplementation on Se levels and oxidative stress, as well as to verify whether Se supplementation improves patient performance in cognitive tests.

## 2. Materials and Methods

### 2.1. Protocol and Registration

The protocol for this systematic review was registered in the International Prospective Register of Systematic Reviews (PROSPERO; CRD42021240649) and was carried out based on the Pattern of Reporting Systematic Review and Meta-Analysis (PRISMA) guideline [26]. The acronym PICO, which corresponds to P = patient or population, I = intervention or indicator, C = comparison or control, and O = outcome, was used to define the guiding question [27,28]. Therefore, in relation to the guiding question of this systematic review and meta-analysis, the acronym PICO corresponds to: P = patients with AD or MCI; I = supplementation with organic or inorganic Se, isolated or in combination with other compounds; C = patients without Se supplementation or who received placebo; and O = improvement in cognitive performance and/or Se availability.

### 2.2. Search Strategy

The Cochrane Library, SciELO, Scopus, and PubMed databases were used in this systematic review. The databases were searched through titles and abstracts, using the keywords “selenium supplementation”, “selenium”, “neuroinflammation”, “Alzheimer’s disease”, and “mild cognitive impairment”, and the Boolean operators “OR” and “AND” (Supplementary Table S1). It is noteworthy that all searches for this study began on December 2020 and finished on March 2021.

### 2.3. Eligibility Criteria

This systematic review included studies that met the following criteria: patients with AD or MCI supplemented with Se (organic or inorganic) or Se plus other nutrients. The exclusion criteria included book chapters, absence of Se supplementation, systematic reviews, other neurodegenerative diseases, and in vitro or animal studies.

### 2.4. Data Extraction

The articles selected for this systematic review were analyzed and classified using the Mendeley Library (Elsevier BV, Amsterdam, The Netherlands). The paper selection process was performed by six reviewers divided into pairs (MEP and FS; JVS and MEAG; and GSV and ILK), who, in the first stage, independently read the titles and abstracts of the papers in relation to the eligibility criteria; any discrepancies were solved by a seventh reviewer (CSO). In the second stage of the selection process, the full text of the potentially eligible records was read. The data extraction included publication year, pathology type (AD or MCI), the country where the study was conducted, design of the study, total number of participants, and the variables that were extracted and selected according to the tests of interest to the topic and the criteria of the systematic review. In addition, the data extracted from the total number of participants in the selected articles included interventions, which divided the participants into two experimental groups (intervention and control). It is worth mentioning that when there was more than groups intervention and control group, this was also presented. Data were extracted from the groups regarding intervention parameters, intervention exposure time, number of participants in the groups, age, number of female and male participants, tests performed, analyzed variables, and results. The data were organized in an Excel 2016 spreadsheet (Microsoft Corp, Washington, WA, USA).

### 2.5. Quality Assessment

Quality assessment was performed using the Cochrane Risk of Bias for Randomized Trials (RoB 2) tool, which is recommended for assessing the risk of bias in randomized trials included in Cochrane reviews. The RoB 2 tool is structured around a fixed set of five domains: domain 1 assesses the randomization process; domain 2 assesses deviations from intended interventions; domain 3 assesses missing results data; domain 4 assesses outcome measurements; and domain 5 assesses the selection of the reported results. Within each domain, there are several questions that aim to obtain information about the study characteristics that are relevant to the risk of bias. A proposal for judging the risk of bias arising from each domain was generated by an algorithm, based on the answers to the questions. As such, the judgment may be “Low” or “High” risk of bias, or it may express “Some concerns” [29].

### 2.6. Statistical Analysis

The meta-analysis was performed using the Review Manager (RevMan) software version 5.3, and the obtained results were considered significant when *p* < 0.05. Random model effects were analyzed, and heterogeneity was evaluated using Higgins inconsistency analyses (I^2^), Cochrane’s Q test, and Tau^2^.

## 3. Results

### 3.1. Selection of Papers

**Figure 1:**
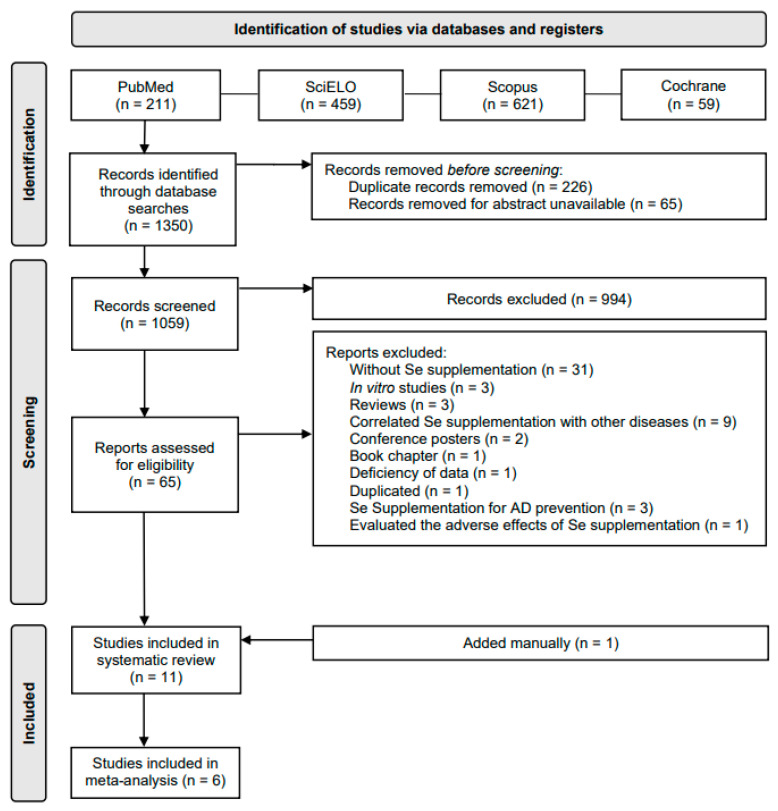
PRISMA flow diagram.

In this study, 1350 scientific papers were collected from four electronic databases. Of these, 226 were duplicates, in 65 documents the abstract was unavailable, and 1049 documents were excluded because they did not meet the eligibility criteria; hence, 65 documents were assessed for eligibility. Of these, 31 did not test Se supplementation, 3 were in vitro studies, 3 were reviews, 9 correlated Se supplementation with other diseases, 2 were conference posters, 1 was a book chapter, 1 had a deficiency of data, 1 was duplicated, 3 reported Se supplementation for AD prevention, and 1 evaluated the adverse effects of Se supplementation; in addition, 1 paper was manually included because it fulfilled the inclusion criteria but it had not been collected through the database searches described above. This resulted in a total of 11 papers that were included in this systematic review: 4 of these papers explored only Se supplementation, and 7 explored Se plus other nutrients supplementation; of those 11 papers, 6 were used in the meta-analysis (Figure 1).

### 3.2. Characteristics of the Systematically Selected Papers

**Figure 2:**
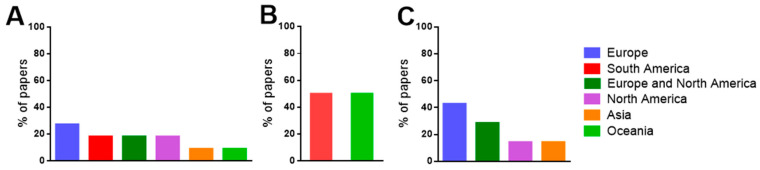
(**A**) Continents where the studies were carried out in all papers included in this systematic review; (**B**) continents where the studies were carried out in papers that performed only Se supplementation; and (**C**) continents where the studies were carried out in papers that performed Se plus other nutrients supplementation.

The 11 papers systematically selected for this study comprised research conducted on five continents: South America (Brazil), North America (USA), Europe (The Netherlands, Germany, Belgium, Spain, Italy, France, and United Kingdom), Asia (Iran), and Oceania (Australia). Most of the papers were from Europe (27.3%), followed by papers from South America (18.2%), Europe and North America (18.2%), North America (18.2%), Asia (9.1%), and Oceania (9.1%) (Figure 2A). Regarding the studies of only Se supplementation, 50% of them were carried out in Oceania and the other 50% in South America (Figure 2B). In contrast, in studies of Se plus other nutrients supplementation, 42.8% were carried out in Europe and North America, and the remainder were carried out in North America (28.6%), Europe (14.3%), and Asia (14.3%) (Figure 2C).

The papers included in this systematic review were outlined as: randomized clinical trials (18.2%); phase IIa randomized control trials (9.1%); randomized, double-blind, and placebo-controlled trials (27.3%); randomized double-blind clinical trials (18.2%); randomized, double-blind, controlled, and multicenter trials (9.1%); randomized, controlled, double-blind, and parallel-group trials (9.1%); and randomized, double-blind, and controlled clinical trials (9.1%) (Table 1 and Table 2). The only Se supplementation studies were outlined as: randomized clinical trial, double-blind, and placebo-controlled pilot studies (40.0%); randomized clinical trials (40.0%); and phase IIa randomized control trials (20.0%) (Table 1). The Se plus other nutrients supplementation studies were outlined as: randomized, double-blind, and placebo-controlled trials (16.6%); randomized double-blind clinical trials (33.3%); randomized, double-blind, controlled, and multicenter trials (16.6%); randomized, controlled, double-blind, and parallel-group trials (16.6%); and randomized, double-blind, and controlled clinical trials (16.6%) (Table 2).

### 3.3. Patient Characteristics

Among the studies exploring supplementation only with Se that were included in this systematic review, 60% included patients with AD and 40% patients with MCI. Regarding sex, the prevalence of female patients was evident: 77 females versus 40 males; there were 49 females with MCI and 28 females with AD. The age range of the patients with MCI was 77.3–79.1 years old, whereas that of patients with AD 68.7–78.8 years old (Table 1).

Among the studies of Se plus other nutrients supplementation included in this systematic review, 87.5% of the participants had AD, and 12.5% had MCI. In terms of sex, the prevalence of females was also evident, with a total of 393 female patients versus 382 male patients. The age range of the patients with MCI was 70.5–88.8 years old, whereas that of patients with AD was 69.1–86.5 years old (Table 2).

### 3.4. Selenium Supplementation

**Figure 3:**
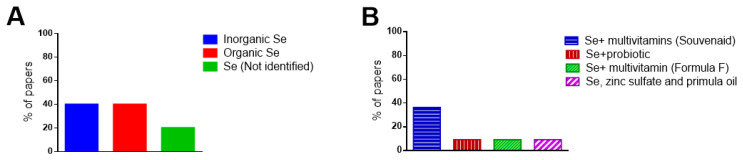
(**A**) Forms of Se supplementation in the paper that evaluated only the Se supplementation; (**B**) Other compounds that are tested in supplementation (Se plus other nutrients).

In the systematically selected papers that evaluated the effects of only Se supplementation, some studies performed supplementation with the inorganic form of Se (40%), others with its organic form (40%), and others did not specify the form of Se used (20%) (Figure 3A).

In studies where Se was administered along with other nutrients, Se was combined with a multivitamin called Souvenaid (containing eicosapentaenoic acid, docosahexaenoic acid, choline, uridine monophosphate, vitamin E, vitamin C, vitamin B12, vitamin B6, and folic acid), with probiotics (containing *L. acidophilus*, *B. bifidum*, and *B. longum*), with a multivitamin called Formula F (containing carnosine, thiamine, riboflavin, nicotinamide, vitamin B6, folic acid, cyanocobalamin, vitamin C, vitamin E, coenzyme Q10, beta carotene, L-cysteine, and *G. biloba*), and with zinc sulfate and primula oil (Figure 3B).

The time of supplementation in the studies that evaluated only Se supplementation was 24 (80.0%) and 12 (20.0%) weeks. In the Se plus other nutrients studies, the period of supplementation was 24 (81.8%), 20 (9.1%), and 12 (9.1%) weeks (Table 1 and Table 2).

### 3.5. Improvement of Se Levels

**Figure 4:**
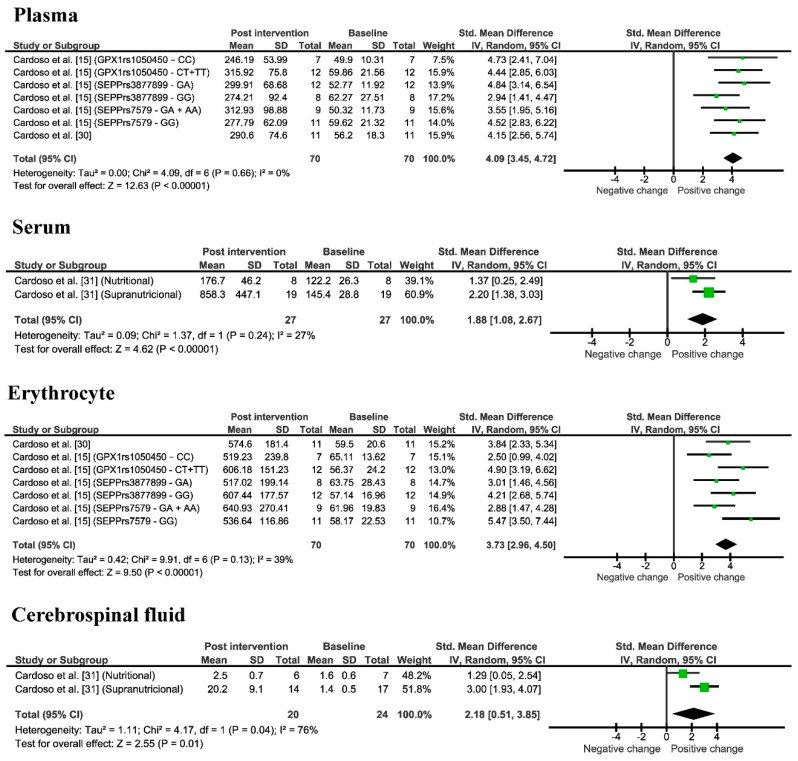
Meta-analysis of Se levels in plasma, serum, erythrocyte, and CSF observed in only selenium supplementation studies pre and post Se supplementation. AD: Cardoso et al. [31]/MCI: Cardoso et al. [30]; Cardoso et al. [15].

In the systematically selected papers, Se levels were measured in different tissues, such as in plasma and erythrocytes in patients with MCI, and in serum and cerebrospinal fluid (CSF) in patients with AD (Table 1 and Table 2). In the only Se supplementation studies, 40.0% of the selected papers analyzed Se in plasma, 20.0% in serum, 20.0% in erythrocytes, and 20.0% in CSF. The range of Se in plasma before supplementation was 49.9–62.3 µg/L and increased to 246.2–315.9 µg/L after Se supplementation; the range of Se in serum was 122.2–145.4 µg/L and increased to 176.7–858.3 µg/L after Se supplementation; the range of Se in erythrocytes was 59.5–65.1 µg/L and increased to 517.0–640.9 µg/L after Se supplementation; finally, the range of Se in the CSF was 1.4–1.6 µg/L and increased to 2.5–20.2 µg/L after Se supplementation. In addition, it was possible to observe in Table 1 that the control group (not supplemented) showed no variation, or even a decrease in Se levels. In fact, the meta-analysis (Figure 4) revealed that Se supplementation significantly increased Se levels by an average of 4.09 (3.45, 4.72) times in plasma (*p* < 0.00001), 1.88 (1.08, 2.67) in serum (*p* < 0.00001), 3.73 (2.96, 4.50) in erythrocytes (*p* < 0.00001), and 2.18 (0.51, 3.85) in CSF (*p* = 0.01).

For the Se plus other nutrients studies, 42.9% of the systematically selected papers analyzed Se levels in plasma, while the other studies (57.1%) did not analyze Se levels. The Se plasma concentration before supplementation was 86.8 µg/L and increased to 102.6–110.5 µg/L after Se supplementation (Table 2). These results were presented as medians, and it was not possible to perform the meta-analysis for this parameter.

### 3.6. Oxidative Stress

**Figure 5:**
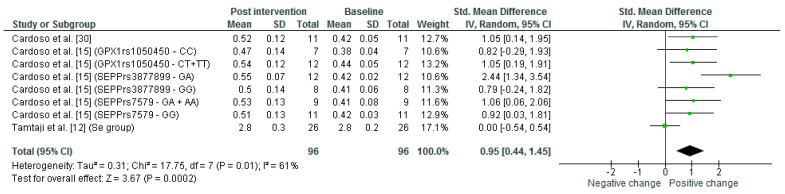
Meta-analysis of MDA levels in plasma in only selenium supplementation studies pre and post Se supplementation. Tamtaji et al. [12] is the only study that underwent treatment for 12 weeks in the other studies the Se supplementation underwent 24 weeks. AD: Tamtaji et al. [12]; MCI: Cardoso et al. [30]; Cardoso et al. [15].

Regarding the oxidative stress analysis, 40% of the studies that evaluated only Se supplementation measured the malondialdehyde (MDA) levels in plasma. It was observed that the range of plasma MDA levels before Se supplementation was 0.4–0.5 μmol/L, whereas after supplementation it was 0.5–0.6 μmol/L (Supplementary Table S2). The meta-analysis (Figure 5) revealed that after Se supplementation MDA levels increased significantly by an average of 0.95 (0.44, 1.45) times (*p* = 0.0002). Regarding the papers included in the Se plus other nutrients supplementation, 28.6% analyzed MDA levels in plasma; before supplementation, the range of plasma MDA levels was 1.2–2.6 μmol/L whereas after supplementation it was 1.5–2.7 μmol/L (Supplementary Table S2). It was not possible to perform the meta-analysis of this parameter because some results were presented as medians.

**Figure 6:**
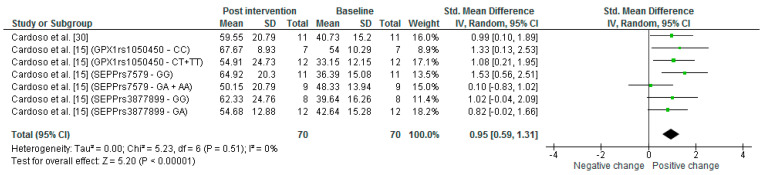
Meta-analysis of GPX activity in erythrocyte observed in Se supplementation group in MCI patients comparing pre and post Se supplementation [15,30].

GPX activity in erythrocytes was analyzed in 40.0% of the papers from the only Se supplementation studies. The GPX activity mean before supplementation ranged from 33.2–54.0 U/gHb, and this range increased to 50.2–67.7 U/gHb after Se supplementation (Supplementary Table S2). In fact, the meta-analysis (Figure 6) showed that GPX activity increased significantly by an average of 0.95 (0.59, 1.31) times (*p* = 0.00001).

### 3.7. Cognitive Performance

In the included papers that realized only Se supplementation, the cognitive tests performed were Controlled Oral Word Association Test—Verbal fluency (COWAT), constructional praxis, Mini-Mental State Examination (MMSE), and Alzheimer’s Disease Assessment Scale-Cognitive subscale (ADAS-Cog). In the Se supplemented group, it is possible to observe that the MMSE score increased from 10.4–19.7 (before Se supplementation) to 19.5–20.0 (after Se supplementation); in COWAT before supplementation, the ranges were 12.8–25.0 and after 14.1–20.0; in ADAS-Cog the score before supplementation was 19.0 and after the supplementation increased to 22.3; and in constructional praxis test, the score before supplementation was 7.7 and after supplementation increased to 9.2. It was not possible to perform the meta-analysis because in some papers the standard deviation was not presented.

In Se plus other nutrients, the cognitive tests performed were the Anomalous Sentences Repetition Test (ASTR), Colored Progressive Matrices (CPM), Digit Copying Test (DCT), Graded Naming Test (GNT), Cambridge Cognitive Examination (CAMCOG), MMSE, ADAS-Cog, and Neuropsychological Test Battery (NTB). In the Se plus other nutrients supplemented group, it is possible to observe that the MMSE score increased from 9.4–23.8 (before supplementation) to 10.4–24.3 (after supplementation); in ADAS-Cog the scores before supplementation were 1.6–25.9 and after supplementation were 1.7–25.9 (Table 2). The meta-analysis showed no statistical difference in the MMSE and ADAS-Cog tests (Supplementary Figure S1).

### 3.8. Risk of Bias

Using the Rob 2 tool, it was possible to determine the risk of bias for the systematically selected papers. Thus, among the studies selected for this systematic review, approximately 36.3% had a high risk of bias, 36.3% had some concerns about the risk of bias, and 27.2% demonstrated a low risk of bias. Regarding the high risk of bias, this was mainly due to domain 2, which deals with deviations from the intended interventions, probably because, in some of the selected studies, there was a lack of information about interventions and how they were applied to the participants. Regarding the bias with some concerns, this was mainly due to domain 4 which is related to the measurement of the outcome, probably due to the influence on the results due to possible knowledge of the interventions received by the participants in some of the studies, or because of the lack of sufficient analyses or inappropriate measurement of these in other studies (Supplementary Figure S2).

## 4. Discussion

Se is an essential element for the maintenance of mammalian life and a component of selenoproteins, which are part of the endogenous antioxidant system. The brain is particularly dependent on Se supply, and is spared from Se deficiency [1,7]. Moreover, Se is important for the protection against oxidative stress [37] and inflammatory processes [38]. Neurodegenerative diseases, such as AD, are characterized by increased oxidative stress and neuroinflammation, which may be due to the accumulation of β-amyloid peptides [7,39,40]. Interestingly, Cardoso et al. [41] demonstrated that patients with AD had significantly lower Se levels in plasma, erythrocytes, and serum, compared to age-matched healthy controls. Similarly, through a systematic review and meta-analysis, Reddy et al. [42] showed a decrease in circulatory, erythrocyte, and CSF Se levels in patients with AD. For the first time, our study demonstrated, through a systematic review and meta-analysis, the possible benefits of Se supplementation on Se levels in patients with MCI or AD, as well as on markers of oxidative stress and on cognitive test performance.

### 4.1. Improvement of Se Levels

According to the Food and Nutrition Board at the Institute of Medicine of the National Academies, USA, it is recommended that people aged >14 years ingest 55 μg Se/day [43]. Importantly, the normal blood Se range for adults is 70–130 µg/L [7], and the daily Se ingestion threshold should not exceed 400 μg because high doses of Se can be toxic to the body [7,30]. Se is already found in the human diet; therefore, supplementation should be performed at appropriate doses, as needed [7,44]. The ideal levels of Se were based on Se levels in the blood, which are associated with maximal GPX activity in adult humans [45,46].

In our study, we observed that in many of the studies that evaluated Se levels before Se supplementation, AD and MCI individuals had Se levels below the ideal blood Se levels [15,30,31]. After supplementation, Se levels increased in various tissues. In fact, the meta-analysis revealed an increase in plasma, serum, erythrocytes, and CSF Se levels. Cardoso et al. [30] pointed out that there is a correlation between Se levels and GPX activity. However, the use of supranutritional Se supplementation must be taken with caution, given Cardoso et al. [31] demonstrated that Se supplementation was associated with some slight side effects. Thus, further studies are needed to determine if the benefits of Se supplementation outweigh any potential side effects.

### 4.2. Oxidative Stress

MDA is one of the products of lipid peroxidation and can be used as a marker of this process [47]. Patients with AD have increased levels of MDA in the plasma [48]. In the systematically selected papers reported in our study we observed that MDA levels did not decrease post-Se-supplementation [12,15,30,37]; in fact, in some cases there was even a slight increase [15,30,37]. Cardoso et al. [30] pointed out that this increase in MDA levels after several weeks of Se supplementation may be an adaptive response of the body. Along these lines, Haddad et al. [49] observed that healthy individuals supplemented with walnuts had their serum MDA levels decreased by 7% five hours after supplementation, but no difference in their serum MDA levels was observed 24 h after supplementation.

Although no decrease in plasma MDA levels was observed after Se supplementation, GPX activity, an important antioxidant enzyme [6], increased after Se supplementation, corroborating the increase in Se levels [15,30]. Garlet et al. [11] observed that GPX activity was positively correlated with Se levels in the blood of individuals with AD. Given that human AD-affected brain tissue has low levels of Se [41], and that Se supplementation has been shown to directly interfere with amyloid and iron neurotoxicity through modulation of GPX activity in primary rat hippocampal neurons [50], supplementation with Se and the consequent increase in GPX activity seem to be a sound approach for the maintenance of brain function of individuals affected by AD and MCI.

### 4.3. Cognitive Performance

MMSE is used as a screening test, and also as a tool to evaluate AD progression by assessing spatial and temporal orientation, immediate and recall memory, calculation, comprehension, writing, and drawing; the MMSE test score increases with better patient performance [51]. In the systematically selected papers, we observed that often MMSE was performed only as a baseline test, which, in many cases, prevented us from evaluating whether there was an improvement in MMSE after Se supplementation. Perhaps performing the test more than once would lead to a better analysis of the patient’s cognitive improvement.

Cardoso et al. [31] demonstrated that individuals with AD who received a supranutritional amount of Se demonstrated a subtle but significant improvement in MMSE, which was associated with an increase in CSF Se levels. Moreover, Cardoso et al. [30] observed that individuals with MCI daily supplemented with Se through Brazil nut intake demonstrated positive responses to certain cognitive functions, such as verbal fluency (storage capacity of the semantic memory system, the ability to retrieve information stored in memory, and the processing of executive functions [52]), and constructive praxis (to assess an individual’s motor, visuospatial, and visuoconstructive skills [53]). When Se supplementation was associated with other nutrients, patients with AD also demonstrated a small improvement in memory performance [35,36].

Oxidative stress is involved in cognitive decline, and studies have shown that patients with MCI and AD present higher levels of reactive species, which culminate in the oxidative stress process [41,54]. Se intake can improve normal cellular function by reducing pro-oxidants molecules and by increasing antioxidant levels in the brain [12], thus reducing neurofibrillary aggregations and achieving better cognitive performance.

## 5. Conclusions

Se supplementation is a good alternative for alleviating some of the symptoms of AD and MCI, such as decreased Se levels and GPX activity, and cognitive deficits. In summary, the systematically selected studies demonstrated improvements in these parameters as a result of Se supplementation (Supplementary Table S3). Additional studies should be performed to analyze the long-term effects of Se supplementation, and how the improved parameters behave after Se supplementation is terminated.

## 6. Limitations

The lack of data and disparity in the parameters used made it difficult to carry out this meta-analysis.

## Figures and Tables

**Table 1:** Summary of systematically selected papers (only Se supplementation).

Reference	Country	Pathology	Groups	Study Type	Characteristics of the Participants	Se Levels (μg/L)	Cognitive Tests (Score)
Cardoso et al. [30]	Brazil	MCI	Se supplementation group consumed one Brazil nut daily for 24 weeks.	Randomized clinical trial	Sex Control Female = 6 Male = 3 Se supplementation Female = 8 Male = 3 Age Control 77.6 ± 6.6 Se supplementation 77.7 ± 4.3	Plasma Control Pre: 50.0 ± 15.5 Post: 47.8 ± 11.7 Se supplementation Pre: 56.2 ± 18.3 Post: 290.6 ± 74.6 Erythrocyte Control Pre: 50.8 ± 21.0 Post: 33.5 ± 16.1 Se supplementation Pre: 59.5 ± 20.6 Post: 574.6 ± 181.4	COWAT Control Pre: 16.3 ± 3.7 Post: 14.1 ± 3.9 Se supplementation Pre: 12.8 ± 3.3 Post: 14.1 ± 3.9 Constructional praxis Control Pre: 8.7 ± 2.6 Post: 8.3 ± 2.4 Se supplementation Pre: 7.7 ± 2.3 Post: 9.2 ± 2.2
Cardoso et al. [15]	Brazil	MCI	Se supplementation group consumed one Brazil nut daily for 24 weeks.	Randomized clinical trial	Sex *GPX1-rs1050450* CC Female = 7 Male = 1 CT + CT Female = 7 Male = 5 *SEPP-rs7579* GG Female = 6 Male = 5 GA + AA Female = 7 Male = 1 *SEPP-rs3877899* GG Female = 8 Male = 4 GA Female = 6 Male = 2 Age *GPX1rs1050450* CC 77.3 ± 7.1 CT + TT 77.83 ± 4.1 *SEPPrs7579* GG 77.4 ± 4.4 GA + AA 78.0 ± 6.5 *S3877899* GG 76.7 ± 3.6 GA 79.1 ± 7.2	Plasma *GPX1-rs1050450* CC Pre: 49.9 ± 10.3 Post: 246.2 ± 54.0 CT + CT Pre: 59.9 ± 21.6 Post: 315.9 ± 75.8 *SEPP-rs7579* GG Pre: 59.6 ± 21.3 Post: 277.8 ± 62.1 GA + AA Pre: 50.3 ± 11.7 Post: 312.9 ± 98.9 *SEPP-rs3877899* GG Pre: 52.8 ± 12.0 Post: 299.9 ± 68.7 GA Pre: 62.3 ± 27.5 Post: 274.2 ± 92.4 Erythrocyte *GPX1-rs1050450* CC Pre: 65.1 ± 13.6 Post: 519.2 ± 239.8 CT + TT Pre: 56.4 ± 24.2 Post: 606.2 ± 151.2 *SEPP-rs7579* GG Pre: 58.2 ± 22.5 Post: 536.6 ± 116.9 GA + AA Pre: 61.9 ± 19.8 Post: 640.9 ± 270.4 *SEPP-rs3877899* GG Pre: 57.1 ± 16.9 Post: 607.4 ± 177.6 GA Pre: 63.7 ± 28.4 Post: 517.0 ± 199.1	-
Malpas et al. [25]	Australia	AD	Se nutritional dose (control): 320 μg sodium selenate 3 times a day for 24 weeks. Se supranutritional dose: 10 mg sodium selenate 3 times a day for 24 weeks.	Phase IIa randomized control trial	Sex Control Female = 8 Male = 12 Supranutritional Female = 9 Male = 11 Age Control 71 Supranutritional 70	-	MMSE Control Pre: 20 Post: 19 Supranutritional Pre: 20 Post: 19 ADAS-Cog Control Pre: 22.1 Post: 22.2 Supranutritional Pre:19.7 Post: 22.3 COWAT Control Pre: 29 Post: 28 Supranutritional Pre: 25 Post: 20
Cardoso et al. [31]	Australia	AD	Se nutritional dose (control): 320 μg sodium selenate 3 times a day for 24 weeks. Se supranutritional dose: 10 mg sodium selenate 3 times a day for 24 weeks.	Randomized, double-blind, placebo-controlled pilot study	Sex Nutritional Female = 4 Male = 4 Supranutritional Female = 15 Male = 4 Age Nutritional 73.4 ± 5.5 Supranutritional 69.5 ± 8.3	Serum Nutritional Pre: 122.2 ± 26.3 Post: 176.7 ± 46.2 Supranutritional Pre: 145.4 ± 28.8 Post: 858.3 ± 447.1 Cerebrospinal fluid Nutritional Pre: 1.6± 0.6 Post: 2.5± 0.7 Supranutritional Pre: 1.4 ± 0.5 Post: 20.2 ± 9.1	-
Tamtaji et al. [12]	Iran	AD	Placebo: placebo (starch) for 12 weeks. Selenium: received selenium 200mg/day for 12 weeks.	Randomized, double-blind, controlled clinical trial	Sex Uninformed Age Placebo 78.5 ± 8.0 Selenium 78.8 ± 10.2	-	MMSE Placebo Pre:9.3 ± 4.1 Post: 9.1 ± 4.4 Selenium Pre:9.9 ± 4.0 Post:10.4 ± 4.2

MMSE: Mini Mental State Examination; COWAT: Controlled Oral Word Association Test—Verbal fluency; and ADAS-Cog: Alzheimer’s Disease Assessment Scale-cognitive subscale.

**Table 2:** Summary of systematically selected papers (Se plus other nutrients supplementation).

Reference	Country	Pathology	Groups	Study Type	Characteristics of the Participants	Se Levels (μg/L)	Cognitive Test (Score)
Van Rhijn et al. [32]	United Kingdom	MCI	Olive Oil (control): six capsules of olive oil and one placebo tablet for 20 weeks. EPO/Zn/Se: six primrose oil capsule (500 mg) and one tablet (200 mg zinc sulphate and 1 mg sodium selenite) for 20 weeks.	Randomized, double-blind, placebo-controlled trial	Sex Olive Oil Female = 11 Male = 4 EPO/Zn/Se Female = 12 Male = 3 Age Olive Oil 83.4 ± 5.4 EPO/Zn/Se 78.7 ± 8.2	-	ASTR Olive Oil Pre: 100.4 ± 17.1 Post: 106.9 ± 19.0 EPO/Zn/Se Pre: 72.6 ± 28.8 Post: 87.5 ± 36.7 CPM Olive Oil Pre: 17.2 ± 6.2 Post: 18.4 ± 9.0 EPO/Zn/Se Pre: 12.2 ± 6.7 Post: 15.8 ± 8.6 GNT Olive Oil Pre: 9.4 ± 7.8 Post: 10.6 ± 8.5 EPO/Zn/Se Pre: 7.2 ± 5.2 Post: 9.8 ± 5.4 DCT Olive Oil Pre: 63.8 ± 14.9 Post: 69.8 ± 18.8 EPO/Zn/Se Pre: 61.2 ± 20.0 Post: 69.7 ± 22.2 CAMCOG Olive Oil Pre: 65.1 ± 17.3 Post: 67.8 ± 17.7 EPO/Zn/Se Pre: 62.50 ± 16.60 Post: 68.30 ± 17.70
Cornelli [33]	United States	AD	Placebo: 500 mg of fructose and flavoring one ampoule/day for 24 weeks. Formula F: Formula F * containing 27.5 µg of Se one ampoule/day for 24 weeks.	Randomized double-blind clinical trial	Sex Placebo Female = 15 Male = 10 Formula F Female = 14 Male = 9 Age Placebo 74 ± 4.9 Formula F 75 ± 4.2	-	MMSE Placebo Pre: 23.9 ± 1.0 Post: 24.2 ± 1.3 Formula F Pre: 23.2 ± 1.1 Post: 24.3 ± 1.4
Scheltens et al. [34]	The Netherlands, Germany, Belgium, United Kingdom and United States	AD	Control: 125 mL tetrapackages without actives once a day for 24 weeks. Active: 125 mL tetrapackages with Souvenaid ** containing 60 µg of Se once a day for 24 weeks.	Randomized, double-blind, controlled, multicenter trial	Sex Control Female = 54 Male = 52 Active Female = 52 Male = 54 Age Control 73.3 ± 7.8 Active 74.1 ± 7.2	-	MMSE Control Pre: 24.0 ± 2.5 Post: 24.0 ± 3.4 Active Pre: 23.8 ± 2.7 Post: 24.1 ± 3.5 ADAS-Cog Control Pre: 25.5 ± 8.8 Post: 25.8 ± 7.8 Active Pre: 25.9 ± 7.6 Post: 25.9 ± 7.7
Scheltens et al. [35]	The Netherlands, Germany, Belgium, Spain, Italy, and France	AD	Control: 125 mL tetrapackages without actives once a day for 24 weeks. Active: 125 mL tetrapackages with Souvenaid ** containing 60 µg of Se once a day for 24 weeks.	Randomized, controlled, double-blind, parallel-group trial	Sex Control Female = 65 Male = 64 Active Female = 62 Male = 68 Age Control 73.2 ± 8.4 Active 74.4 ± 6.9	-	NTB Control Pre: 0.1 ± 0.1 Post: 0.1 ± 0.5 Active Pre: 0.1 ± 0.8 Post: 0.2 ± 0.4 ADAS-Cog Control Pre: 1.2 ± 1.5 Post: 1.4 ± 1.4 Active Pre: 1.6 ± 1.7 Post: 1.7 ± 1.6
Shah et al. [36]	United States	AD	Control: 125 mL tetrapackages without actives once a day for 24 weeks. Active: 125 mL tetrapackages with Souvenaid ** containing 60 µg of Se once a day for 24 weeks.	Randomized, double-blind clinical trial	Sex Control Female = 135 Male = 127 Active Female = 139 Male = 126 Age Control 76.9 ± 8.2 Active 76.6 ± 8.2	-	ADAS-Cog Control Pre: 23.4 ± 9.3 Post: 24.4 ± 10.9 Active Pre: 23.9 ± 9.6 Post: 25.4 ± 11.6
Rijpma et al. [37]	The Netherlands, Germany, Belgium, United Kingdom and United States	AD	Control: 125 mL tetrapackages without actives once a day for 24 weeks. Active: 125 mL tetrapackages with Souvenaid ** containing 60 µg of Se once a day for 24 weeks.	Randomized double-blind, multicenter, controlled trial	Sex Control Female = 54 Male = 52 Active Female = 52 Male = 54 Age Control 73.3 ± 7.8 Active 74.1 ± 7.2	Plasma Control Pre: 86.8 Post: 78.9 Active Pre: 86.8 Post: 102.6	-
Rijpma et al. [37]	The Netherlands, Germany, Belgium, Spain, Italy, and France	AD	Control: 125 mL tetrapackages without actives once a day for 24 weeks. Active: 125 mL tetrapackages with Souvenaid ** containing 60 µg of Se once a day for 24 weeks.	Randomized double-blind, multicenter, controlled trial	Sex Control Female = 65 Male = 64 Active Female = 62 Male = 68 Age Control 73.2 ± 8.4 Active 74.4 ± 6.9	Plasma Control Pre: 86.8 Post: 86.8 Active Pre: 86.8 Post: 110.5	-
Tamtaji et al. [12]	Iran	AD	Placebo: placebo (starch) for 12 weeks. Selenium: 200 mg of Se plus probiotic *** every day for 12 weeks.	Randomized, double-blind, controlled clinical trial	Sex Uninformed Age Placebo 78.5 ± 8.0 Probiotic plus selenium 76.2 ± 8.1	-	MMSE Placebo Pre: 9.3 ± 4.1 Post: 9.1 ± 4.4 Selenium plus probiotic Pre: 9.4 ± 3.5 Post:10.9 ± 3.8

EPO/Zn/Se: primrose oil, zinc sulphate and sodium selenite; ASTR: Anomalous Sentences Repetition Test; CPM: Colored Progressive Matrices; DCT: Digit Copying Test; GNT: Graded Naming Test; CAMCOG: Cambridge Cognitive Examination; MMSE: Mini-Mental State Examination; ADAS-Cog: Alzheimer’s Disease Assessment Scale-cognitive subscale; and NTB: Neuropsychological Test Battery. * Formula F contain: 100 mg Carnosine, 1.4 mg Thiamine (B1), 1.6 mg Riboflavin (B2), 18 mg Nicotinamide (B3), 2 mg Pyridoxine (B6), 200 μg Folic acid (B9), 1 μg Cyanocobalamin (B12), 30 mg Vitamin C, 20 mg Vitamin E, 10 mg Coenzyme Q10, 800 RE β -Carotene, 27.5 μg Selenium, 10 mg L-cysteine, and 25 mg *Ginkgo biloba*. ** Souvenaid contains: 300 mg Eicosapentaenoic acid, 1200 mg Docosahexaenoic acid, 106 mg Phospholipids, 400 mg Choline, 625 mg Uridine monophosphate, 40 mg Vitamin E, 80 mg Vitamin C, 60 μg Selenium, 3 mg Vitamin B12, 1 mg Vitamin B6, and 400 μg Folic acid. *** Probiotic contains: 2 × 10^9^ CFU of *L. acidophilus*, *B. bifidum*, and *B. longum.*

## Data Availability

Not applicable.

## Supplementary Materials

The following supporting information can be downloaded at: https://www.mdpi.com/article/10.3390/nu14153205/s1, Table S1: Search strategy for each electronic database; Table S2: MDA levels and GPX activity; Table S3: Main findings in papers included in this systematic review; Figure S1: Cognitive Performance (MMSE and ADAS-cog) meta-analysis in selenium plus other compounds group in patients with AD; Figure S2: Risk of bias.
