# Supplementary material for: Effects of Selenium Supplementation in Patients with Mild Cognitive Impairment or Alzheimer’s Disease: A Systematic Review and Meta-Analysis
Source: Nutrients. 2022 Aug 5;14(15):3205. doi: 10.3390/nu14153205 (PMC9370215; doi:10.3390/nu14153205)
Supplement: Supplementary file 1 [file nutrients-14-03205-s001.zip › nutrients-1821010-supplementary.pdf]

Supplementary Table S1. Search strategy for each electronic database.

Database	Strategy
**PubMed (title/abstract)**	selenium supplementation OR selenium AND neuroinflammation OR Alzheimer's disease OR mild cognitive impairment
**SciELO (abstract)**	selenium supplementation OR selenium AND neuroinflammation OR Alzheimer's disease OR mild cognitive impairment
**Scopus (title/abstract)**	selenium supplementation OR selenium AND neuroinflammation OR Alzheimer's disease OR mild cognitive impairment
**Cochrane Library (title/abstract)**	selenium supplementation OR selenium AND neuroinflammation OR Alzheimer's disease OR mild cognitive impairment

Supplementary Table S2. MDA levels and GPX activity.

Reference	MDA (µmol/L)	GPX (U/gHb)
**Only Se supplementation**		
Cardoso et al. [30]	Control<br>Pre: 0.5±0.1<br>Post: 0.5±0.2<br><br>Se supplementation<br>Pre: 0.4±0.1<br>Post: 0.5±0.1	Control<br>Pre: 44.0±17.2<br>Post: 42.5±13.1<br><br>Se supplementation<br>Pre: 40.7±15.2<br>Post: 59.6±20.8
Cardoso et al. [15]	***GPX1-rs1050450***<br>CC<br>Pre: 0.4±0.0<br>Post: 0.5±0.1<br>CT+TT<br>Pre: 0.4±0.1<br>Post: 0.5±0.1<br><br>***SEPP-rs7579***<br>GG<br>Pre: 0.4±0.0<br>Post: 0.5±0.1<br>GA+AA<br>Pre: 0.4±0.1<br>Post: 0.5±0.1<br><br>***SEPP-rs3877899***<br>GG<br>Pre: 0.4±0.1<br>Post: 0.5±0.1<br>GA<br>Pre: 0.4±0.0<br>Post: 0.6±0.1	***GPX1rs-1050450***<br>CC<br>Pre: 54.0±10.3<br>Post: 67.7±8.9<br>CT+TT<br>Pre: 33.2±12.2<br>Post: 54.9±24.7<br><br>***SEPP-rs7579***<br>GG<br>Pre: 36.4±15.1<br>Post: 64.9±20.3<br>GA+AA<br>Pre: 48.3±13.9<br>Post: 50.2±20.8<br><br>***SEPP-rs3877899***<br>GG<br>Pre: 39.6±16.3<br>Post: 62.3±24.8<br>GA<br>Pre: 42.6±15.3<br>Post: 54.7±12.9
Tamtaji et al. [12]	Placebo<br>Pre: 2.7±0.4	-

	Post: 2.7±0.4<br><br><u>Selenium</u><br>Pre: 2.8±0.2<br>Post: 2.8±0.3	
**Se plus other nutrients supplementation**		
Rijpma et al. [37]	<u>Control</u><br>Pre: 1.2±0.5<br>Post: 1.5±0.6<br><br><u>Active</u><br>Pre: 1.2±0.6<br>Post: 1.5±0.5	-
Tamtaji et al. [12]	<u>Placebo</u><br>Pre: 2.7±0.4<br>Post: 2.7±0.4<br><br><u>Probiotic plus selenium</u><br>Pre: 2.7±0.3<br>Post: 2.6±0.2	-

Supplementary Table S3. Main findings in the papers included in this systematic review.

Reference	Main findings
**Only Se supplementation**	
Cardoso et al. [30]	The intake of one Brazil nut daily restored Se levels in plasma and in erythrocytes of older adults with MCI, as well as caused an improvement in verbal fluency and constructive praxis.
Cardoso et al. [15]	The intake of one Brazil nut daily restored the Se levels and GPX activity in erythrocytes of older adults with MCI.
Malpas et al. [25]	AD individuals supplemented with Se (sodium selenate) had less atrophy of the hippocampus when compared to the control group.
Cardoso et al. [31]	AD individuals supplemented with Se (sodium selenate) showed an increase in serum and CSF Se levels, as well as had significant improvement in MMSE score.
Tamtaji et al. [12]	AD individuals supplemented with Se presented a favorable improvement in MMSE score and reduced glutathione levels.
**Se plus other nutrients supplementation**	
Van Rhijn et al. [32]	MCI patients supplemented with Se plus other nutrients had an improved performance in ASTR, CPM, GNT, and DCT cognitive tests.
Cornelli 2010 [33]	AD individuals supplemented with Se plus other nutrients had an improvement in MMSE score and reduced glutathione levels.
Scheltens et al. [34]	AD individuals supplemented with Se plus other nutrients had an improvement in the delayed verbal recall task.
Scheltens et al. [35]	AD individuals supplemented with Se plus other nutrients had an improvement in memory function domain Z-score of the Neuropsychological test Battery.
Shah et al. [36]	AD individuals supplemented with Se plus other nutrients did not present improvement in the cognitive parameters evaluated.

Rijpma et al. [37]	AD individuals supplemented with Se plus other nutrients had an improvement in plasma Se levels.
Rijpma et al. [37]	AD individuals supplemented with Se plus other nutrients had an improvement in plasma Se levels.
Tamtaji et al. [12]	AD individuals supplemented with Se plus other nutrients presented a favorable improvement in MMSE score, reduced glutathione and MDA levels.

ASTR: Anomalous Sentences Repetition Test; CPM: Colored Progressive Matrices; GNT: Graded Naming Test; DCT: Digit Copying Test

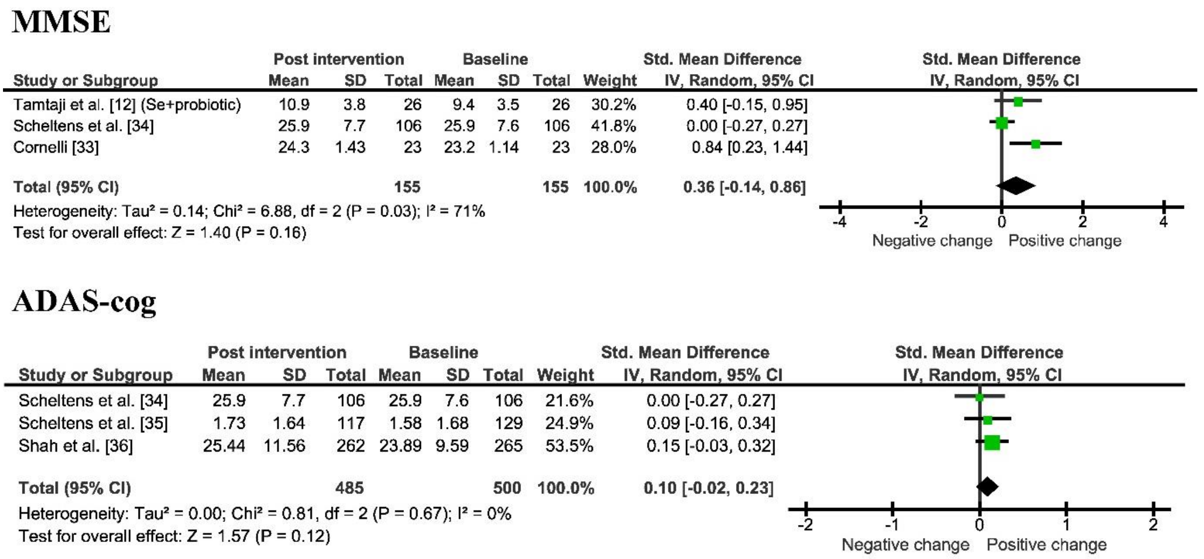

Supplementary Figure S1. Cognitive Performance (MMSE and ADAS-cog) meta-analysis in selenium plus other nutrients supplementation studies in patients with AD, comparing before and after treatment.

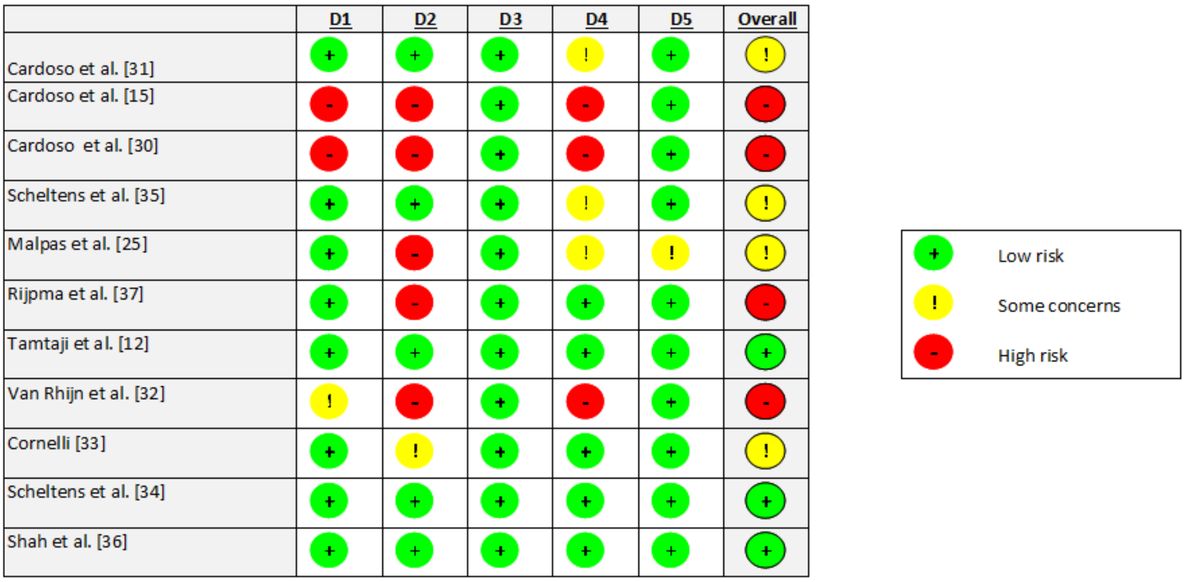

Supplementary Figure S2. Risk of bias. The domains analyzed were randomization process (D1), deviations from the intended interventions (D2), missing outcome data (D3), measurement of the outcome (D4), and selection of the reported result (D5).
